# Ursolic acid protects monocytes against metabolic stress-induced priming and dysfunction by preventing the induction of Nox4^☆^

**DOI:** 10.1016/j.redox.2014.01.003

**Published:** 2014-01-11

**Authors:** Sarah L. Ullevig, Hong Seok Kim, Huynh Nga Nguyen, William S. Hambright, Andrew J. Robles, Sina Tavakoli, Reto Asmis

**Affiliations:** a Department of Kinesiology, Health, and Nutrition, University of Texas at San Antonio, United States; b Department of Clinical Laboratory Sciences, University of Texas Health Science Center, San Antonio, United States; c Department of Biochemistry, University of Texas Health Science Center, San Antonio, United States; d Department of Cellular and Structural Biology, University of Texas Health Science Center, San Antonio, United States; e Department of Pharmacology, University of Texas Health Science Center, San Antonio, United States; f Department of Radiology, University of Texas Health Science Center, San Antonio, United States

**Keywords:** Grx, glutaredoxin, GSH, reduced glutathione, HFD, high-fat diet, HG, high d-glucose, LDL, low-density lipoprotein, MAPK, mitogen-activated protein kinase, MKP-1, MAPK phosphatase-1, MCP-1, monocyte chemoattractant protein-1, Nox4, NADPH oxidase 4, OA, oleanolic acid, PSSG, protein–glutathione mixed disulfide, ROS, reactive oxygen species, UA, ursolic acid, Ursolic acid, Nox4, Monocyte, *S*-glutathionylation, Atherosclerosis

## Abstract

**Aims:**

Dietary supplementation with ursolic acid (UA) prevents monocyte dysfunction in diabetic mice and protects mice against atherosclerosis and loss of renal function. The goal of this study was to determine the molecular mechanism by which UA prevents monocyte dysfunction induced by metabolic stress.

**Methods and results:**

Metabolic stress sensitizes or “primes” human THP-1 monocytes and murine peritoneal macrophages to the chemoattractant MCP-1, converting these cells into a hyper-chemotactic phenotype. UA protected THP-1 monocytes and peritoneal macrophages against metabolic priming and prevented their hyper-reactivity to MCP-1. UA blocked the metabolic stress-induced increase in global protein-*S*-glutathionylation, a measure of cellular thiol oxidative stress, and normalized actin-*S*-glutathionylation. UA also restored MAPK phosphatase-1 (MKP1) protein expression and phosphatase activity, decreased by metabolic priming, and normalized p38 MAPK activation. Neither metabolic stress nor UA supplementation altered mRNA or protein levels of glutaredoxin-1, the principal enzyme responsible for the reduction of mixed disulfides between glutathione and protein thiols in these cells. However, the induction of Nox4 by metabolic stress, required for metabolic priming, was inhibited by UA in both THP-1 monocytes and peritoneal macrophages.

**Conclusion:**

UA protects THP-1 monocytes against dysfunction by suppressing metabolic stress-induced Nox4 expression, thereby preventing the Nox4-dependent dysregulation of redox-sensitive processes, including actin turnover and MAPK-signaling, two key processes that control monocyte migration and adhesion. This study provides a novel mechanism for the anti-inflammatory and athero- and renoprotective properties of UA and suggests that dysfunctional blood monocytes may be primary targets of UA and related compounds.

## Highlights

• Ursolic acid protects monocytes from metabolic stress-induced dysfunction or “metabolic priming” by preventing the induction of Nox4.
• Ursolic acid restores p38 MAP kinases activation and chemotactic responses to MCP-1 in monocytes exposed to metabolic stress.
• Ursolic acid prevents increased protein *S*-glutathionylation, including actin-*S*-glutathionylation, in metabolically stressed monocytes.
• Ursolic acid prevents MKP-1 degradation and restores MKP-1 activity in metabolically stressed monocytes.

## Introduction

Ursolic acid (UA), a cyclic triterpenoid, is an anti-inflammatory phytochemical widely distributed within the plant kingdom and found in medicinal and traditional herbs, as well as a large number of fruits [1], [2], [3]. Initially studied for its anti-cancer properties, UA induces apoptosis in cancer cells and reduces tumor growth [1]. More recently, UA's anti-inflammatory properties have been studied in the context of metabolic disorders and UA is emerging as a potential preventative and therapeutic agent for metabolic diseases. UA has been reported to affect a multitude of enzymes involved in inflammatory processes, including, but not limited to, cyclooxygenase 2 (COX2) [4], NF-ĸB [5], [6], and nitric oxide synthase (NOS) [4], [7], [8]. In disease-specific animal models, UA administration was shown to protect and preserve the functionality of various organs including liver [9], [10], kidney [11], [12], [13], pancreas [14], skeletal muscle [15], and brain [16], [17]. UA showed beneficial effects in rodent models of hypertension [18], obesity [15], and diabetes [13], [19]. We recently showed that UA protects diabetic mice against diabetic complications, including atherosclerosis [13]. However, the molecular mechanisms underlying these beneficial properties of UA are largely unknown.

Atherosclerosis is characterized by chronic infiltration of inflammatory cells, particularly monocytes, into the subendothelial space in the vascular wall [20]. Chemoattractant-stimulated monocyte recruitment and transmigration into the vessel wall dominate all stages of atherosclerosis and play a fundamental role in the initiation and progression of atherosclerotic lesions. Within lesions, monocyte-derived macrophages orchestrate the continuous infiltration of inflammatory cells and the remodeling of the vessel wall, thereby maintaining a chronic state of inflammation [20]. Chronic inflammation and oxidative stress are hallmark features of metabolic diseases, including atherosclerosis, and drive disease progression [21]. We recently reported that metabolic stress transforms monocytes into a proatherogenic phenotype, resulting in their hyper-responsiveness to chemoattractants, a process we coined monocyte priming [22]. Monocyte priming correlates with both increased monocyte chemotaxis and recruitment *in vivo* and accelerated atherosclerotic lesion formation, suggesting that monocyte priming by metabolic stress may be a novel, fundamental mechanism underlying atherosclerosis and other chronic inflammatory diseases [22]. We demonstrated that monocyte priming is mediated by NADPH oxidase 4 (Nox4)-induced thiol oxidative stress and the subsequent dysregulation of redox sensitive signaling pathways [22], [23], [24]. We went on to show that Nox4 induction was both necessary and sufficient to promote metabolic priming in monocytes [22].

Nox4 is one among the seven members of the NAPDH oxidase family whose function is to transport electrons across a membrane to produce reactive oxygen species (ROS) [25]. Unlike the majority of Nox proteins, which produce superoxide, Nox4 appears to primarily produce hydrogen peroxide (H_2_O_2_) [26], [27], [28]. In response to physiological stimuli, Nox4 generates H_2_O_2_ and activates signaling pathways, such as insulin [29] and epidermal growth factor signaling [30], through the oxidation of specific protein thiols. Protein thiols can undergo oxidation to various oxidation products, including *S*-glutathionylated thiols, i.e., mixed disulfide bonds between protein thiols and glutathione [31]. Protein-*S*-glutathionylation is an important post-translational modification in redox signaling and can inhibit or activate protein function [32], [33], and even target proteins for degradation [23], [34]. We recently found that increased actin-*S*-glutathionylation in response to metabolic stress increases actin turnover in monocytes, which appears to contribute to enhanced monocyte adhesion to endothelium and accelerated monocyte migration and tissue infiltration [22], [23]. Furthermore, we found that in response to metabolic stress, mitogen-activated protein kinase phosphatase 1 (MKP-1) is glutathionylated, targeting MKP-1 for proteasomal degradation. MKP-1 *S*-glutathionylation results in the hyperactivation of MAPK signaling pathways that control monocyte adhesion and migration [22], [23], [24].

Current prevention strategies and treatments for metabolic and chronic inflammatory diseases focus mainly on reducing or preventing inflammation and oxidative stress. Due to their relatively low cost and low toxicity, phytochemicals may provide an attractive alternative to current approaches in disease prevention and management. A number of compounds have shown promise for reducing or even reversing symptoms of diseases characterized by chronic inflammation [35], [36], [37]. We recently reported, in a mouse model of diabetic complications, that dietary UA reduces monocyte dysfunction and protects against accelerated atherosclerosis and kidney injury [13], but the underlying mechanisms are unknown. In this study, we provide evidence that UA protects blood monocytes from metabolic priming and dysfunction by inhibiting the induction of Nox4 and reducing cellular protein-*S*-glutathionylation, specifically, *S*-glutathionylation of two important redox signaling proteins essential for monocyte adhesion and migration, actin and MKP-1. Based on these data, we propose a novel mechanism of action that may explain many of the anti-inflammatory properties of UA. Our study highlights the therapeutic potential of UA and related compounds.

## Materials and methods

### Chemicals and reagents

Unless stated otherwise, chemicals were purchased from Sigma-Aldrich, St. Louis, MO, cell culture reagents from Gibco^®^ Invitrogen, Grand Island, NY, and all primers and supplies for qPCR were purchased from Invitrogen, Grand Island, NY.

#### Monocyte priming

Monocyte priming was induced as described previously [22]. Briefly, human THP-1 monocytes (ATCC, Manassas, VA) at 1–2×10^6^ cells/ml were cultured at 37 °C for 20 h in RPMI-1640 (Hyclone and Cellgro^®^) containing, 10% fetal bovine serum (FBS), 5.5 mM d-glucose, 2% Glutamax, 1% sodium pyruvate (Cellgro^®^), 1% penicillin/streptomycin (Cellgro^®^), 1% HEPES, 0.1% β-2-mercaptoethanol, and supplemented with either phosphate buffered saline (PBS) or freshly isolated native human LDL (100 µg/ml in PBS) plus d-glucose (high glucose, 20 mM). l-glucose does not increase monocyte priming [22]. For selected experiments, peritoneal macrophages were collected from C57BL/6 mice by peritoneal lavage and purified by negative selection using antibody-coated magnetic beads (Dynabeads^®^ mouse pan B (B220) and Dynabeads^®^ mouse pan T (Thy 1.2)). This procedure routinely increased the macrophage content of the isolate from approximately 40% CD68-positive cells to greater than 95% CD68 positive cells. Purified macrophages were cultured in Teflon bags under non-adherent conditions [38], and primed for 24 h in complete RPMI-1640 medium supplemented with human LDL (100 µg/ml in PBS) plus d-glucose (20 mM, HG)

#### LDL isolation

LDL was isolated by KBr-gradient ultracentrifugation from pooled plasma from healthy blood donors and purified by gel-filtration chromatography, filter-sterilized and characterized as described previously [39], [40].

#### Monocyte chemotaxis assay

THP-1 monocytes or purified peritoneal macrophages were primed with HG+LDL for 20–24 h in the presence of either vehicle (dimethyl sulfoxide, DMSO, ≤0.1%) or UA, then loaded into the upper wells of a 48-well modified Boyden chamber (NeuroProbe, Gaithersburg, MD). The lower wells contained either vehicle or 2 nM MCP-1 (R&D Systems, Minneapolis, MN). A 5 µm polyvinyl pyrrolidone-free polycarbonate filter membrane was layered between the upper and lower chambers, and the chamber was incubated for 2 h for THP-1 monocytes or 3 h for peritoneal macrophages at 37 °C and 5% CO_2_. The membrane was washed and cells removed from the upper side of the filter. Transmigrated cells were stained with Diff-Quik^®^ Set (Dade Behring, Newark, DE) and counted in four–five separate high power fields at 400× magnification under a light microscope.

#### Western blot analysis

Cells were washed with ice-cold PBS and lysed on ice in RIPA lysis buffer (50 mM Tris–HCl, pH 7.5, 150 mM NaCl, 1% Nonidet P-40, 0.1% SDS, 0.5% sodium deoxycholate) with protease inhibitor and/or phosphatase inhibitors. Aliquots with equal amounts of protein were loaded and separated on an 8% or 10% SDS-PAGE gel. Proteins were transferred to polyvinylidene difluoride membranes (PVDF, Millipore, Billerica, MA) and probed using specific antibodies. The following antibodies were used: Nox4 [41] (available from Epitomics, 3174-1, Burlingame, CA), Anti-glutathione antibody: Millipore (MAB5310, Billerica, MA), p38/p38-phospho: Cell Signaling (9212S and 9211S, respectively, Danvers, MA) and MKP-1: Santa Cruz (SC-370, Santa Cruz, CA), actin: Santa Cruz (SC1615), Grx-1: R&D systems (AF3399, Minneapolis, MN). Bands were detected by chemiluminescence on a KODAK Image Station 4000MM (Carestream, Rochester, NY). To control for sample loading, blots were subsequently stripped and re-probed for total p38 or actin.

### Reverse transcription quantitative polymerase chain reaction (RT-qPCR)

Briefly, total RNA was extracted using the PureLink RNA Mini Kit and quantified using a NanoDrop spectrophotometer (ThermoScientific, Rockford, IL). Total RNA (1 μg) was synthesized into cDNA using the Maxima First Strand cDNA Synthesis Kit (ThermoScientific, Asheville, NC). Taqman probes were used for all genes (Grx-1: Hs00829752_g1, Nox2: Hs01553393_m1, GAPDH:Hs99999905_m1) using the cycling conditions described by the manufacturer. No amplification was detected in no-template control wells. Gene expression levels were normalized to GAPDH and mRNA fold-change relative to control wells was calculated using the ΔΔCt method [42]. Four biological replicates and three technical replicates were performed.

#### MKP-1 activity assays

MKP-1 activity was determined with a modification of the commercially available MalachiteGreen-based PTP assay (Millipore, Billerica, MA). Briefly, to assess MKP-1-specific PTP activity, lysates were analyzed both in the absence and presence of 40 µM sanguinarine (SG), a specific inhibitor of MKP-1 (34). SG-sensitive PTP activity was attributed to MKP-1. Briefly, assays were initiated by adding 10 µl of phosphotyrosine peptide substrate to cell extracts (2 µg protein) diluted in 20 mM Tris–HCl (pH 7.5), 150 mM NaCl, 1% NP-40 and warmed to 30 °C. The reaction was stopped after 10 min. MKP-1 activity was assayed spectrophotometrically as the amount of inorganic phosphate released using a VersaMax (Molecular Devices, Sunnyvale, CA). Phosphate released by MKP-1 was quantified from a standard curve prepared with known amounts of KH_2_PO_4_.

#### Statistics

Data were analyzed using ANOVA (SigmaStat, Systat Software, San Jose, CA). Data were tested for use of parametric or nonparametric post hoc analysis, and multiple comparisons were performed by using the Least Significant Difference method. All data are presented as mean±SE of at least 3 independent experiments unless stated otherwise. Results were considered statistically significant at the *P*<0.05 level.

## Results

### Ursolic acid protects monocytes against metabolic priming

**Fig. 1:**
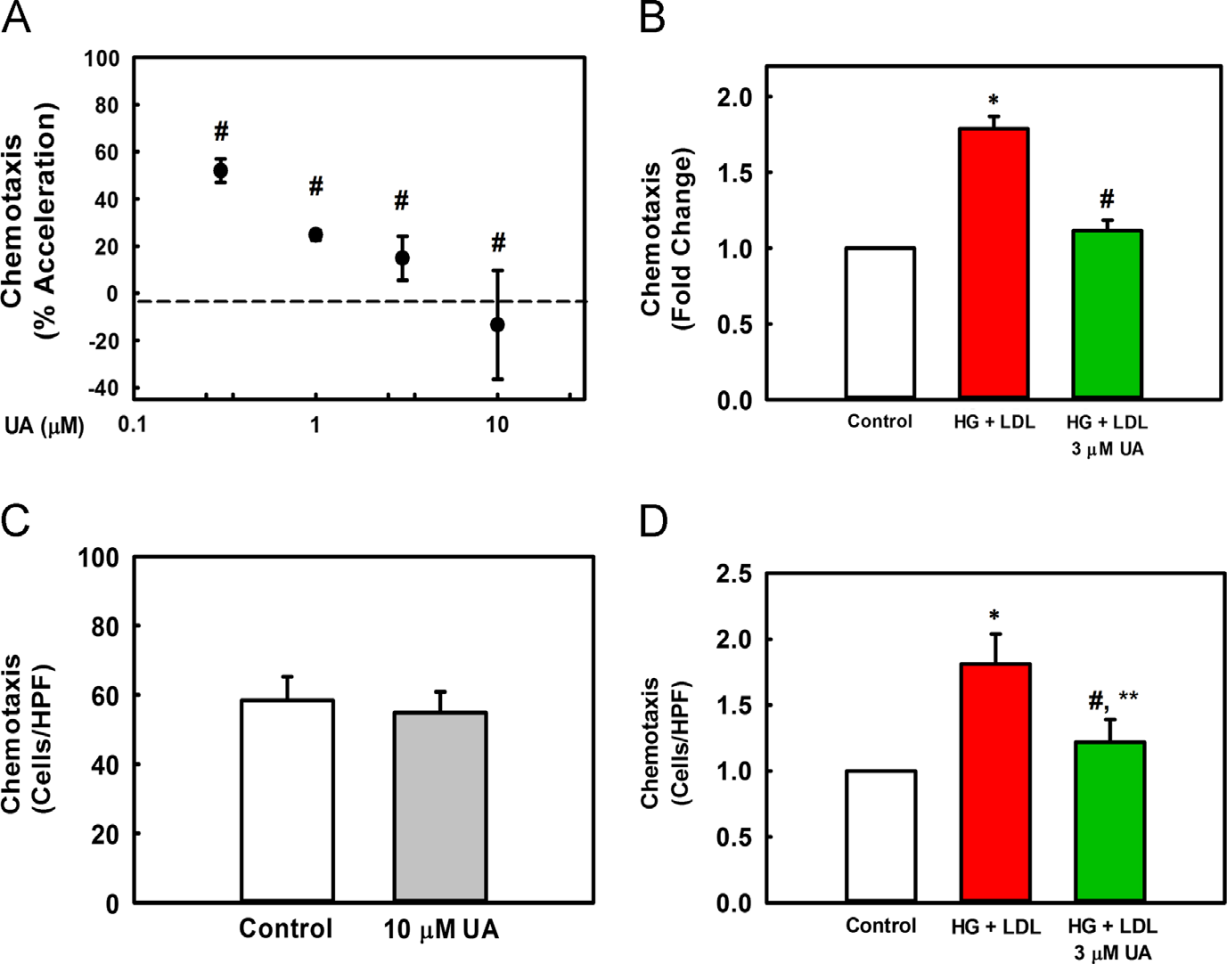
UA attenuates metabolic stress-induced acceleration of monocyte chemotaxis in response to MCP-1. (A) THP-1 cells cultured in RPMI 1640 medium (5 mM glucose, 10% FBS) were treated for 20 h with HG (20 mM d-glucose) and native LDL (100 µg/ml) in the presence of 0, 0.3, 1.0, 3.0 or 10 µM UA or vehicle (DMSO). The supernatant was removed and cells were resuspended in 0.1% FBS-containing RPMI medium. Cells were then transferred into a multi-well Boyden chamber and stimulated with 2 nM MCP-1 for 2 h. Migrated cells were counted in 4 high-power fields (HPF) per well, 4 wells for each condition. Data were normalized to the accelerating effect of metabolic stress on chemotaxis (“100%”), i.e., values obtained for HG+LDL-primed THP-1 monocytes stimulated with MCP-1 minus values obtained from unprimed THP-1 monocytes stimulated with MCP-1 (“0%”;dotted line). Results are shown as mean from 5 independent experiments±SE; #versus 100% acceleration, *P*=0.038 (0.3 µM), *P*=0.002 (1, 3 µM), *P*<0.001 (10 µM). (B) Chemotaxis was assayed as in (A). The graph depicts the fold change induced by HG+LDL in MCP-1-stimulated chemotaxis (red bar) and by HG+LDL+3 µM UA (green bar) versus unprimed, MCP-1 stimulated control cells (white bar). *n*=5, mean±SE. ^⁎^versus unprimed control (no metabolic stress), *P*<0.001; #versus HG+LDL, *P*=0.002, (C) Chemotaxis was assessed in unprimed cells treated with either vehicle (open bar) or UA (gray bar) as described in (A). Values represent means of 4 HPF counts for control cells, and cells treated with vehicle or 10 µM UA; mean±SE; n=4, *P*=0.712. (D) Chemotaxis was assessed in unprimed mouse peritoneal macrophages (open bars), or macrophages that were metabolically primed (red bars) or metabolically primed in the presence of UA (green bar). ^⁎^versus unprimed control macrophages (no metabolic stress), *P*=0.002; #versus HG+LDL, *P*=0.016; ^⁎^^⁎^versus unprimed control macrophages, *P*=0.30.

Previously, we showed that UA inhibits the priming effect of oxidative stress, i.e. extracellular H_2_O_2,_ on monocyte chemotaxis with a median inhibitory concentration (IC_50_) of 0.45 µM [13]. We also reported that THP-1 monocytes exposed to metabolic stress, i.e. high glucose (HG, 25 mM) plus human LDL (100 µg/ml), shows a similar hypersensitivity to MCP-1 as oxidatively stressed THP-1 monocytes [22]. We therefore tested if UA also protected THP-1 monocytes against chemokine hypersensitivity and dysfunction induced by metabolic stress. UA prevented monocyte priming in a dose-dependent manner (Fig. 1A and B). In the presence of 3 µM UA, monocyte priming was reduced by 83%, and at 10 µM, normal chemotactic responses were restored (Fig. 1A and B). In agreement with our previous studies with H_2_O_2_-treated THP-1 monocytes [13], UA inhibited monocyte priming with an IC_50_ of 0.4 µM, indicating this inhibition may occur through a similar mechanism. Importantly, UA treatment alone did not affect MCP-1-stimulated chemotaxis in unprimed monocytes (Fig. 1C), suggesting that UA targets specific mechanisms or signaling pathways involved in the dysregulation of monocyte migration, but not chemotaxis *per se*.

To confirm that the protective effects of UA were not limited to THP-1 monocytes, we repeated these experiments in purified peritoneal macrophages isolated from C57BL/6 mice. Murine peritoneal macrophages exposed to metabolic stress (HG+LDL) *ex vivo* showed a similar hyper-sensitization to MCP-1-induced chemotaxis as primed THP-1 cells (Fig. 1B and D). Importantly, when UA was present during metabolic priming by HG+LDL, the increased chemotactic responses of peritoneal macrophages were prevented (Fig. 1D).

### Ursolic acid reduces both total protein-*S*-glutathionylation and actin-*S*-glutathionylation induced by metabolic stress

**Fig. 2:**
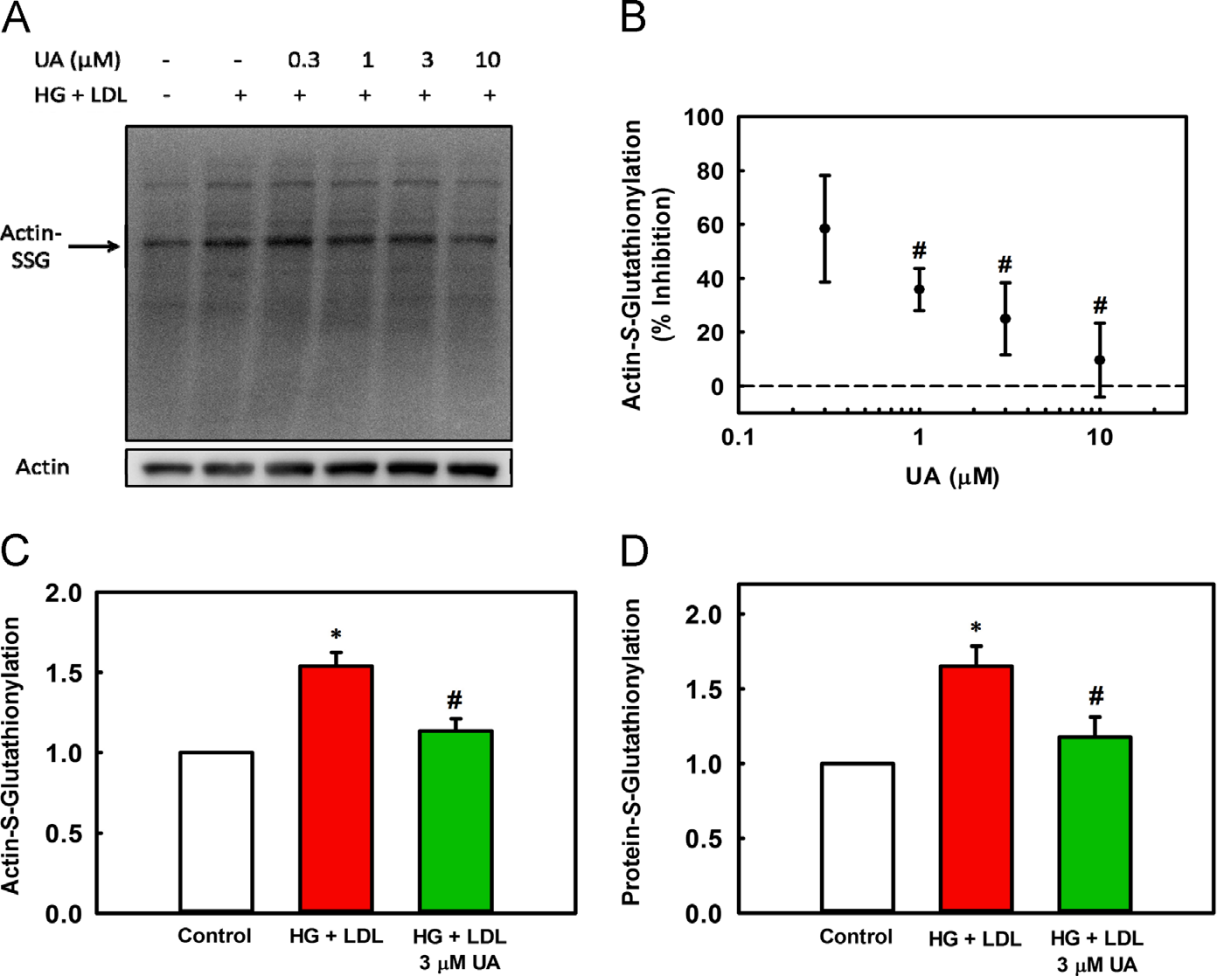
UA reduces actin- and total-*S*-glutathionylation induced by metabolic stress. THP-1 monocytes in RPMI 1640 medium (5 mM glucose, 10% FBS) were treated with 0.3, 1, 3, 10 µM UA or vehicle. HG (20 mM glucose) plus native LDL (100 µg/ml) was present for 20 h where indicated. Cells were lysed in the lysis buffer containing 10 mM NEM. Actin- and protein-*S*-glutathionylation was assessed by Western blot analysis using the anti-glutathione antibody. Western Blot data for actin-*S*-glutathionylation is summarized in A–C. (A) A representative Western Blot is shown. (B) Quantitation by Western blot analysis assessed using an anti-glutathione antibody is shown of actin-S-glutathionylation in response to increasing doses of UA. *n*=4, mean±SE. # versus 100% actin-*S*-glutathionylation, *P*=0.004 (1 µM), *P*=0.003 (3 µM), *P*≤0.001 (10 µM). **(C)** Quantitative data for actin-*S*-glutathionylation and the effects of 3 µM UA. Data is represented as fold change induced by HG+LDL (red bar) and HG+LDL+ 3 µM UA (green bar) versus unprimed control cells (white bar). *n*=3, mean±SE; ^⁎^versus Control, *P*=0.006, # versus HG+LDL, *P*=0.022. (D) Total protein-*S*-glutathionylation was determined by Western blot and the density of the entire lane was measured and normalized to actin. Total protein-*S*-glutathionylation is represented as fold change induced by HG+LDL (red bar) and HG+LDL+3 µM UA (green bar) versus unprimed control cells (white bar). *n*=4, mean±SE, ^⁎^versus control, *P*<0.001, #versus HG+LDL, *P*=0.003.

The dysregulation of monocyte chemotactic responses by metabolic stress (HG+LDL) is mediated by increased cellular protein-*S*-glutathionylation, including the increased *S*-glutathionylation of actin [22], [24]. We now found that UA dose-dependently inhibited actin-*S*-glutathionylation induced by metabolic stress (Fig. 2A and B). At 3 µM UA, hyper-*S*-glutathionylation of actin was reduced by 75% (Fig. 2C). At the same concentration, UA also reduced by 73% total cellular protein-*S*-glutathionylation induced by metabolic priming (Fig. 2D**)**, suggesting that UA targets a protein or a pathway responsible for mediating metabolic stress-induced *S*-glutathionylation of multiple proteins. At 10 µM UA, levels of actin S-glutathionylation were completely normalized to levels seen in healthy control cells (Fig. 2A).

### Ursolic acid does not alter Grx1 mRNA or protein levels

Glutaredoxin-1 (Grx1) is the primary cytosolic enzyme that specifically reduces *S*-glutathionylated proteins in THP-1 monocytes [43]. Overexpression of Grx1 in THP-1 monocytes reduces *S*-glutathionylated proteins and prevents the conversion of monocytes into the proatherogenic primed phenotype [22]. To determine whether Grx1 expression was a target of UA, we measured Grx1 mRNA by quantitative PCR and protein expression by Western Blot. Surprisingly, neither Grx1 mRNA nor protein expression was significantly altered by UA in either primed or unprimed THP-1 monocytes (Supplementary Fig. 1A and B). In unprimed THP-1 monocytes, UA treatment resulted in an increase in Grx1 protein expression (40% increase), but the difference was not statistically significant (*P*=0.073). The inhibitory effect of UA on protein-*S-*glutathionylation in metabolically primed monocytes can therefore not be explained by induction of Grx1.

### Ursolic acid rescues MAPK phosphatase-1 protein degradation and activity

**Fig. 3:**
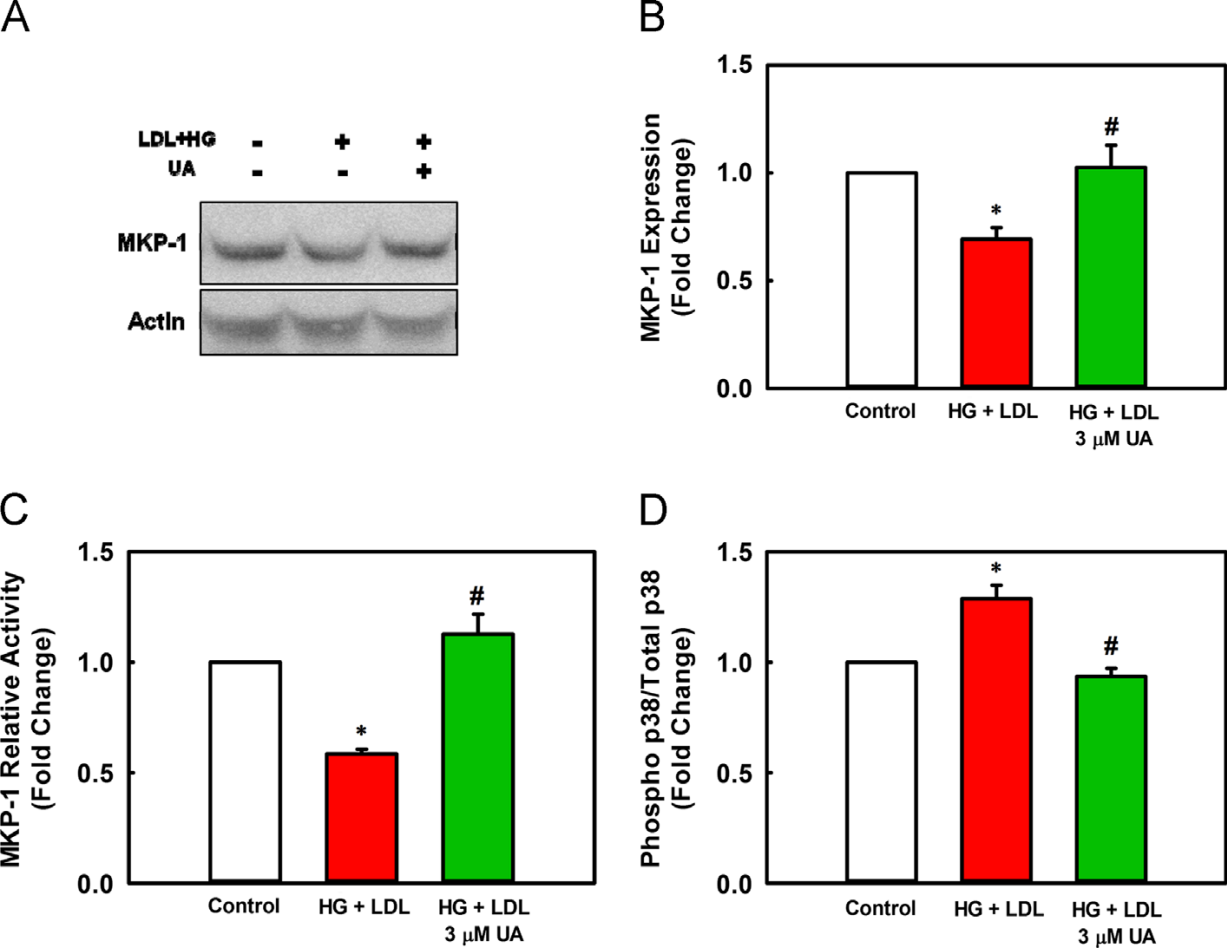
UA rescues MKP-1 protein expression and activity in metabolically primed THP-1 monocytes. THP-1 cells were treated for 20 h with 3 µM UA or vehicle control in the presence of HG+LDL. (A) Representative Western blot MKP-1 protein levels. (B) Quantitation of Western blot analysis. Data was normalized to actin and is shown as mean±SE of 3 independent experiments. ^⁎^versus unprimed control cells (no metabolic stress), *P*=0.017; #versus HG+LDL primed cells, *P*=0.012. (C) MKP-1 phosphatase activity was assessed using a modification of the commercially available Malachite Green-based PTP assay as described under *Material and Methods. n*=3, ^⁎^versus unprimed control cells (no metabolic stress), *P*=0.002; #versus HG+LDL, *P*<0.001. (D) Phospho p38 was measured by Western blot analysis as described in “Material and methods” section. Data was normalized to total p38. *n*=3, mean±SE; ^⁎^versus unprimed control cells (open bar), *P*=0.003, #versus HG+LDL (red bar), *P*<0.001, HG+LDL+3 µM UA (green bar).

MAPK phosphatase-1 (MKP-1) is a redox sensitive phosphatase that regulates the phosphorylation and activity of p38 and Erk proteins [44], [45], [46]. Metabolic priming of monocytes promotes MKP-1-*S*-glutathionylation, resulting in MKP-1 inactivation and subsequent proteasomal degradation [23]. We therefore examined whether UA could protect MKP-1 protein expression and activity in metabolically stressed THP-1 monocytes. At 3 µM, UA prevented the metabolic stress-induced degradation of MPK-1 (Fig. 3A and B) and fully rescued MKP-1 activity in metabolically primed THP-1 monocytes (Fig. 3C).

Loss of MKP-1 activity leads to the hyperactivation of p38, as measured by the phosphorylation of p38, both in resting THP-1 monocytes and in response to MCP-1 stimulation [23]. We therefore determined if UA also prevents the hyperactivation of p38 in metabolically primed THP-1 monocytes. UA normalized p38 phosphorylation to levels found in healthy control cells (Fig. 3D). These data suggest that, under conditions of metabolic stress, UA protects MAPK signaling pathways that control monocyte adhesion and migration, by preventing MKP-1-*S*-glutathionylation, inactivation and degradation.

### Ursolic acid reduces Nox4 protein expression

**Fig. 4:**
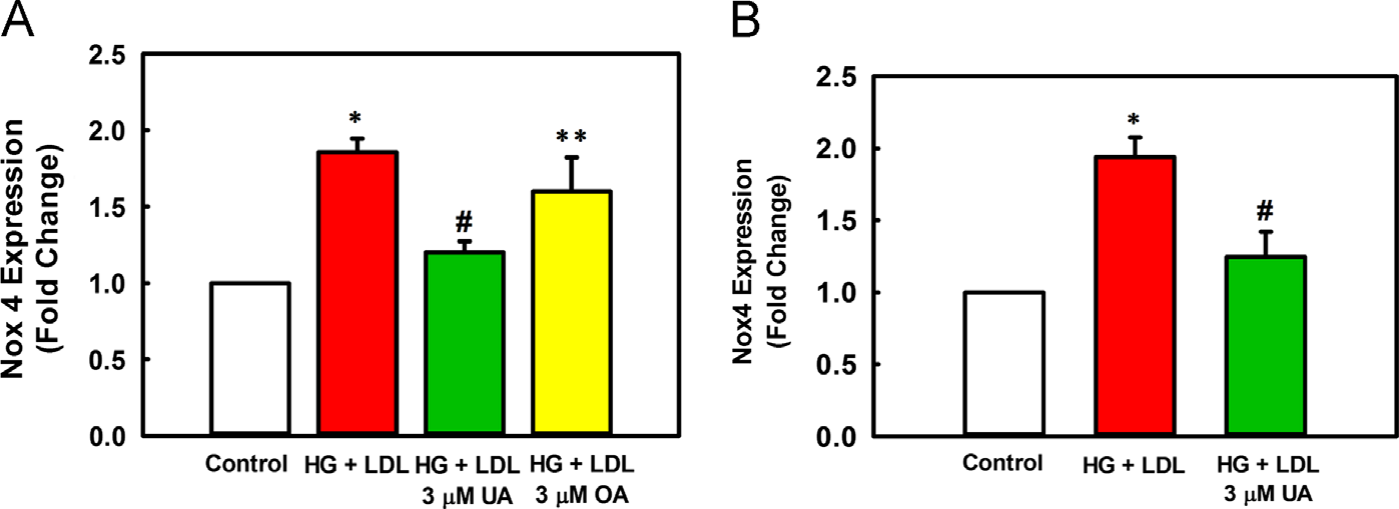
UA prevents Nox4 protein induction by metabolic stress. (A) THP-1 monocytes cultured in RPMI 1640 medium (5 mM glucose, 10% FBS) were treated with 3 µM UA, 3 µM OA, or vehicle for 1 h prior to the addition of glucose (20 mM HG) plus native LDL (100 µg/ml) for an additional 20 h. Nox4 protein expression was determined by Western blot analysis as described under “Material and methods” section. Data was normalized to actin levels. Results are shown as mean±SE of 3–4 independent experiments. ^⁎^versus unprimed control (open bars, no metabolic stress), *P*<0.001; #versus HG+LDL primed cells (red bar), *P*<0.001; ^⁎^^⁎^versus HG+LDL, *P*=0.081. HG+LDL+3 µM UA (green bar) and HG+LDL+3 µM OA (yellow bar). (B) Mouse peritoneal macrophages treated *ex vivo* with HG+LDL, with or without 3 µM UA, for 24 h. Nox4 protein expression was determined as in (A). *n*=3, ^⁎^versus unprimed control (open bars, no metabolic stress), *P*=0.002; #versus HG+LDL primed cells (red bar), *P*=0.008.

Nox4-derived H_2_O_2_ mediates metabolic stress-induced monocyte priming and the *S*-glutathionylation of actin and MKP-1 induced by metabolic stress [22]. Since UA prevented actin-*S*-glutathionylation induced by metabolic stress and rescued MKP-1 degradation and activity without altering Grx-1 expression, we next investigated whether UA is able to prevent the induction of Nox4 we observed in metabolically-primed THP-1 monocytes. Indeed, we found that at 3 µM, UA inhibited the metabolic stress-induced increase in Nox4 protein levels by 77% (Fig. 4A, Supplementary Fig. 2). UA also blocked the induction of Nox4 in metabolically stressed mouse peritoneal macrophages (Fig. 4B).

Oleanolic acid (OA) is a structural isomer of UA that differs only in the position of one methyl group. Despite its structural similarities to UA, OA is 3.5-fold less potent than UA in inhibiting accelerated monocyte chemotaxis induced by metabolic stress (IC_50_ of OA=1.4 µM, data not shown, versus an IC_50_=0.4 µM for UA, Fig. 1A). Here we show that OA was also significantly less potent at blocking metabolic-stress-stimulated Nox4 induction. At 3 µM, OA only inhibits Nox4 induction by 30%, compared to 77% inhibition by UA at the same concentration (Fig. 4A). Both UA and its analog OA appear to protect THP-1 monocytes against metabolic priming by blocking Nox4 protein expression induced by metabolic stress.

Nox2 is the primary Nox isoform found in monocytes and macrophages and is a potential source of ROS that could promote protein-*S*-glutathionylation and contribute to the effects of metabolic stress on monocyte chemotaxis. However, we found no induction of Nox2 in metabolically primed THP-1 monocytes nor did UA have any effect on Nox2 mRNA levels in either control or metabolically primed THP-1 monocytes (Supplementary Fig. 3). This finding confirms our previous report that Nox2 does not mediate thiol oxidative stress and monocyte dysfunction induced by metabolic stress [22].

## Discussion

We recently reported that metabolic stress, *in vitro* and *in vivo,* results in the hyper-sensitization of monocytes to chemoattractants, a process we termed monocyte priming. Metabolic priming of monocytes results in the increased adhesion, accelerated chemotaxis and increase recruitment of monocyte-derived macrophages in response to chemokines [22], [23], [24]. Not only may monocyte priming be involved in atherogenesis, but it also appears to contribute to the acceleration of atherosclerosis and renal injury associated with diabetes [22]. Dietary supplementation with UA prevented the accumulation of inflammatory monocytes in the blood of diabetic mice, reduced *in vivo* monocyte chemotactic activity in these mice, improved renal function, and decreased both plaque size, and macrophage content in atherosclerotic lesions in these mice [13]. These studies suggested that UA may directly target blood monocytes and protect them from metabolic stress-induced priming, preventing them from converting into a proatherogenic, hyper-inflammatory phenotype. The goal of this study was to determine the molecular mechanisms through which UA prevents monocyte dysfunction and thus may exert its anti-atherogenic and renoprotective properties.

**Fig. 5:**
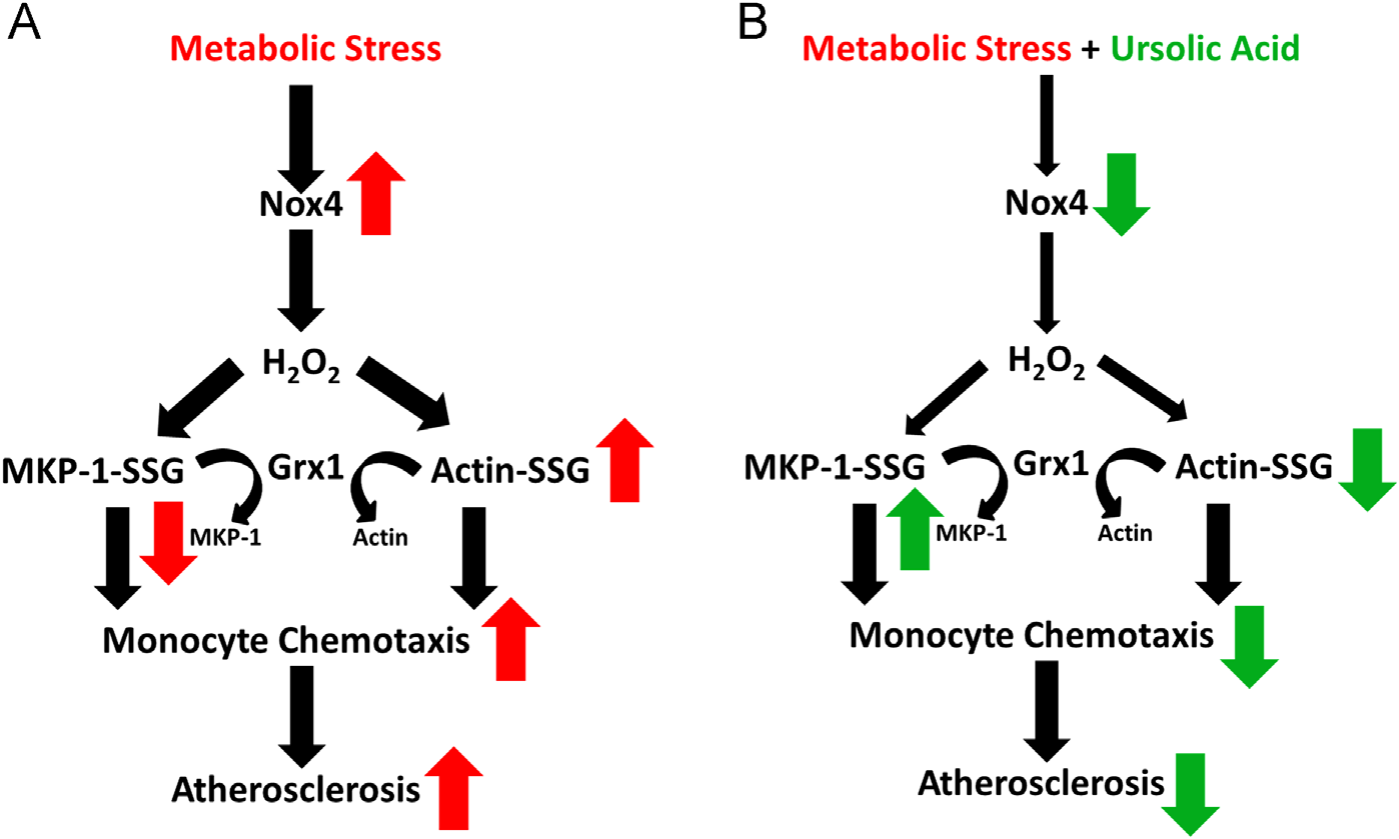
Hypothetical model for the mechanism of action of UA in metabolically primed monocytes. (A) The effects of metabolic stress are indicated by red arrows. Metabolic stress (HG+LDL) increases Nox4 protein expression and Nox4-derived H_2_O_2_ formation [22]. We hypothesize that Nox4-derived H_2_O_2_ promotes increases in total cellular protein-*S*-glutathionylation and the *S*-glutathionylation of actin and MKP-1, two proteins important in monocyte migration and adhesion [22], [23], [24]. The increase in actin and MKP-1 *S*-glutathionylation results in increased monocyte chemotaxis and accelerated macrophage recruitment [22], rate-limiting steps in the development of atherosclerosis. (B) The effects of ursolic acid (UA) are indicated by green arrows. In the presence of ursolic acid, the metabolic stress-induced induction of Nox4 protein expression and subsequent *S*-glutathionylation of MKP-1 and actin are prevented. Normal monocyte migration is restored in the presence of ursolic acid.

Monocyte priming by metabolic stress involves the early induction of Nox4, Nox4-dependent thiol oxidation and the subsequent, persistent protein-*S*-glutathionylation of a large number of proteins, processes which all contribute to the accelerated chemotactic responses to chemokine stimulation (Fig. 5) [22]. Here we report that UA blocked these effects of metabolic stress on both human THP-1 monocytes and murine peritoneal macrophages. Since Nox4 induction is both necessary for metabolic priming and sufficient to induce metabolic priming in monocytes [22], we hypothesized that UA targets Nox4 expression in metabolically primed monocytes. Indeed, we found that UA prevented the induction of Nox4 in metabolically primed monocytes at concentrations that also blocked hyper-*S*-glutathionylation of actin, MKP-1 *S*-glutathionylation and degradation, and the exaggerated chemotactic response of primed monocytes to MCP-1 (Fig. 5). Yet, Nox2 expression levels were not affected by UA, suggesting the inhibitory effect of UA is specific for Nox4 and appears to occur at the transcriptional or translational level, rather than by inhibiting Nox4 activity itself, although further studies are needed to confirm this hypothesis. Our findings are in agreement with a previous study reporting that UA treatment of a human endothelial cell line reduces Nox4 expression [8].

Based on mapped consensus sequences in the Nox4 promoter region, Nox4 transcription may be under the control of several transcription factors, including NF-kB, peroxisome proliferator-activated receptors (PPARs), members of the O subclass of forkhead transcription factors (FOXO), and SMA/MAD related transcription factor (SMAD) [47]. It is possible that UA regulates Nox4 transcription. Many of UA’s anti-inflammatory and anti-tumor effects have been shown to coincide with reduced NF-kB expression and activation [5], [6]. In a liver cell line, UA was reported to increase both PPARα expression and binding of activated PPARα to peroxisome proliferator response elements (PPRE), thereby activating gene transcription [48]. Collectively, these data suggest that UA may prevent Nox4 induction at the transcriptional level by blocking the binding of transcription factors, such as NF-kB, to the Nox4 promoter.

Alternatively, UA may suppress Nox4 expression by inhibiting translational events. Nox4 translation was shown to be regulated by serum [49] and microRNAs [50], including miR-25c [51], miR-145ac [52], miR-23b[53]. It is unclear at this point, whether UA affects any of these translational events, although in a glioblastoma cell line, UA was shown to suppress miR-21 [54]. One regulator of protein synthesis activated by high glucose levels is mTOR. Interestingly, mTOR was reported to be inhibited by UA [55]. This finding suggests that inhibition of mTOR may be another plausible mechanism to explain UA's ability to suppress Nox4 expression induced by metabolic stress. Indeed, we found that the mTOR inhibitor rapamycin reduced Nox4 protein expression in unprimed THP-1 monocytes (unpublished data), suggesting that UA may prevent Nox4 induction and monocyte priming via an mTOR-dependent pathway.

While the exact mechanisms through which UA prevents metabolic stress-induced Nox4 expression remains to be elucidated, the ability of UA to block Nox4 induction, and thus metabolic priming in monocytes, may explain UA’s potent anti-inflammatory properties *in vivo*, including its ability to improve kidney function and block atherosclerotic lesion formation in diabetic mice [13]. It is important to note, that the dosages of UA at which we observed protective effects in cultured human THP-1 monocytes and murine peritoneal macrophages (0.3–3 µM) are well within the range of the UA plasma concentrations reported in published studies (0.1–2.4 µM) [56], [57]. In our hands, diabetic mice fed a diet enriched with 0.2% UA, a dose that suppresses atherosclerotic lesion formation and renal injury, showed UA plasma concentrations that ranged from 0.1 to 0.3 µM (unpublished results), indicating that the UA concentrations we used in our cell culture experiments and the observed benefits are physiologically relevant.

In summary, we found that UA prevents metabolic stress-induced monocyte priming by blocking Nox4 induction, thereby inhibiting the dysregulation of two important thiol redox-sensitive signaling mechanisms involved in monocyte adhesion and migration, i.e. actin turnover and MAPK signaling (Fig. 5). This study provides a novel mechanism of action for UA which may explain its anti-inflammatory and anti-atherogenic properties.

## Funding sources

This work was supported by grants to R.A. from the NIH (AT-006885) and the Morrison Trust (F065300). S.U. was supported by a fellowship from the Translational Science Training (TST) Across Disciplines program at the University of Texas Health Science Center at San Antonio, with funding provided by the University of Texas System's Graduate Programs Initiative.

## Footnotes

Appendix A Supplementary data associated with this article can be found in the online version at doi:10.1016/j.redox.2014.01.003.
