# Supplementary figures and images for: Ursolic acid protects monocytes against metabolic stress-induced priming and dysfunction by preventing the induction of Nox4
Source: Redox Biol. 2014 Jan 11;2:259–66. doi: 10.1016/j.redox.2014.01.003 (PMC3909821; doi:10.1016/j.redox.2014.01.003)

# Supplemental Figure 1

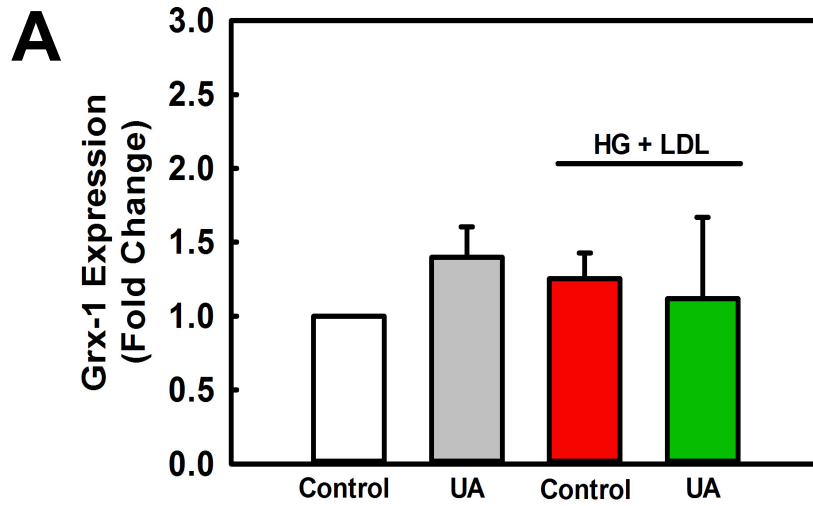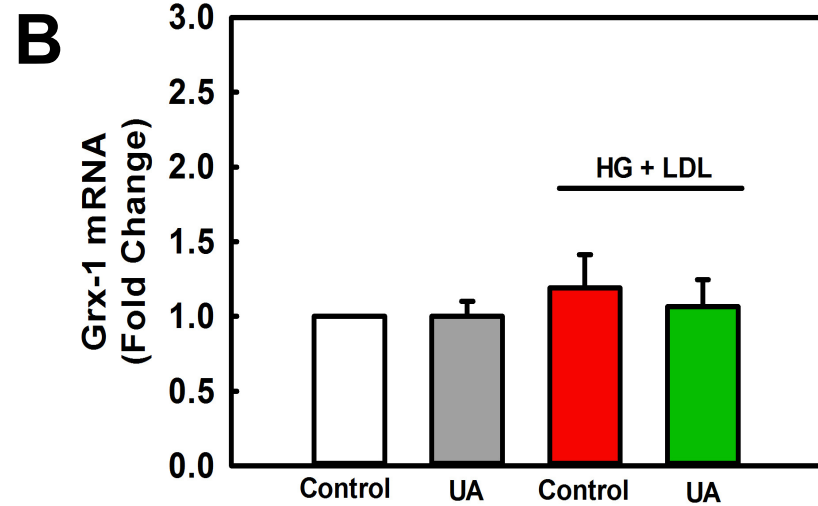

## Supplement 2

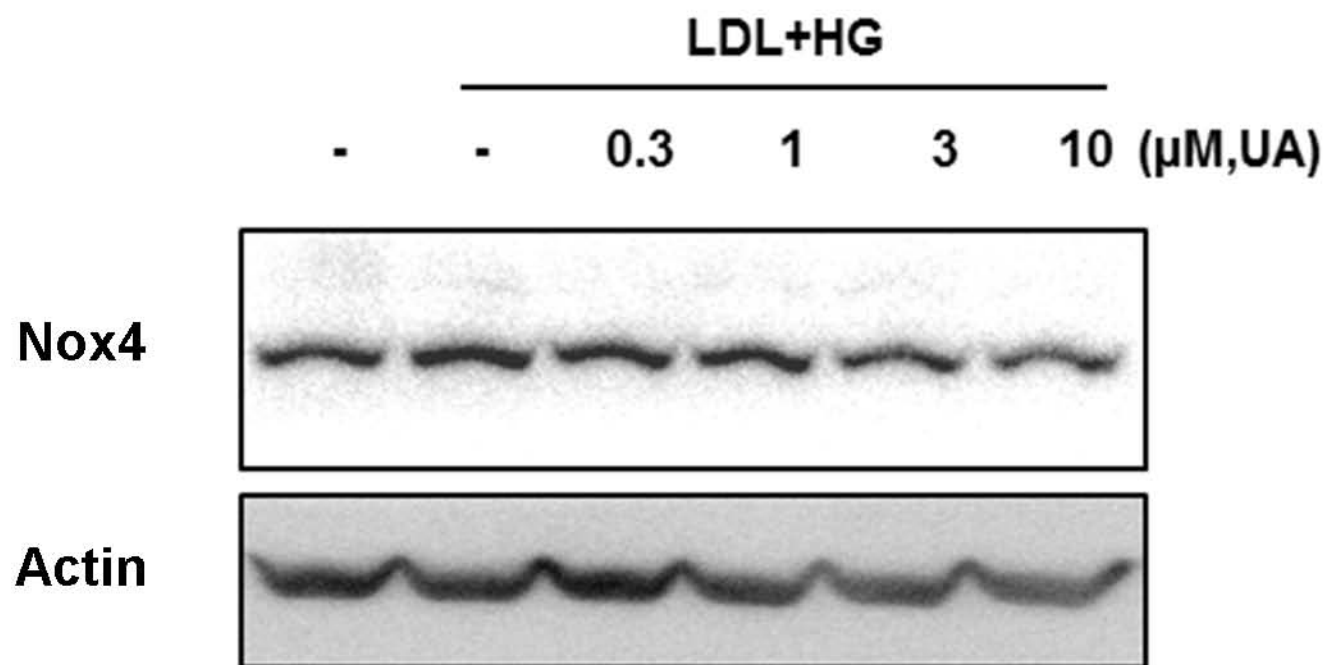

## Supplemental Figure 3

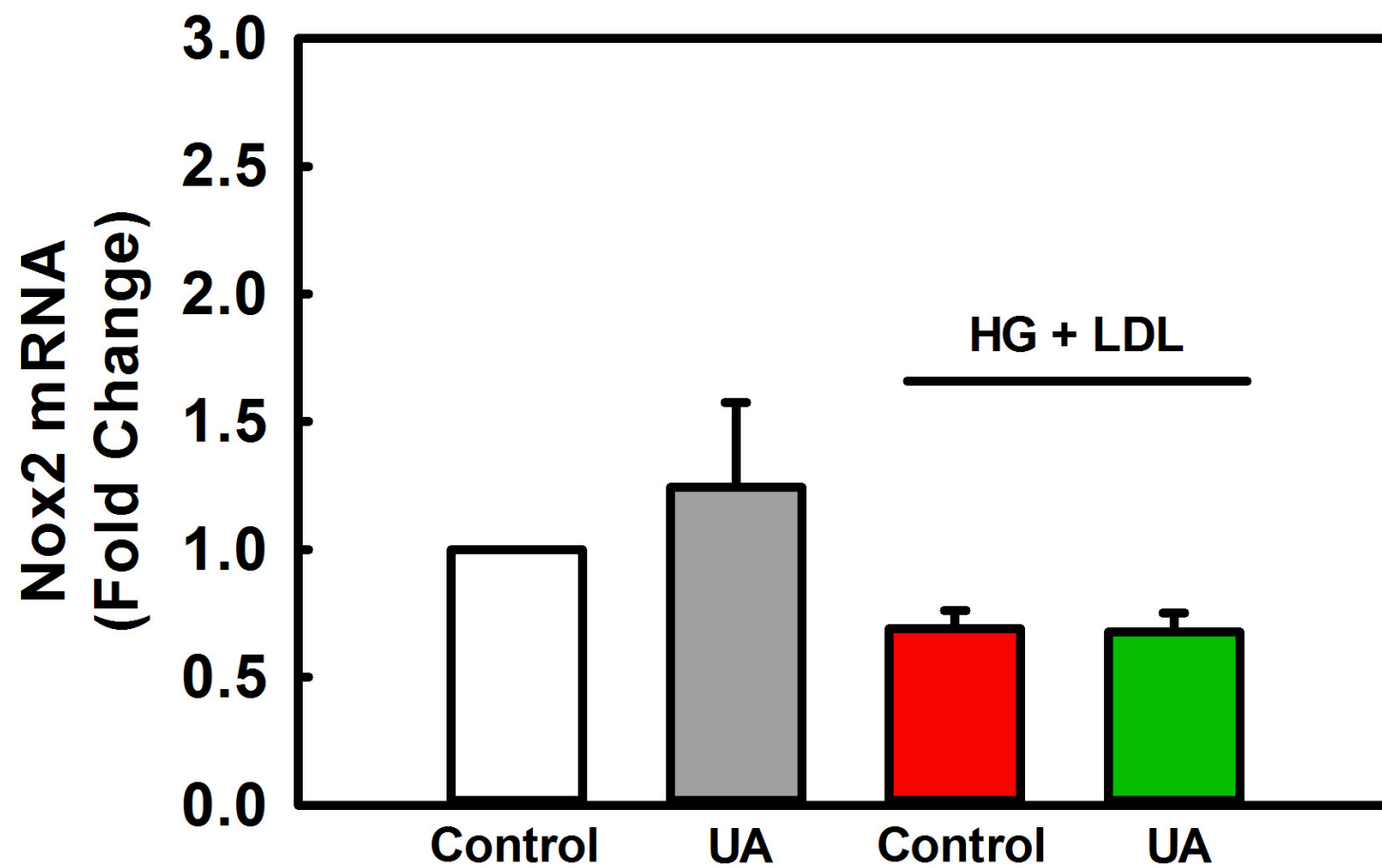

Supplement: Supplementary file 1 — Supplementary material [file mmc1.pdf]
